# Multicenter (FX)_n_/NH_3_ Halogen Bonds (X = Cl, Br and *n* = 1–5). QTAIM Descriptors of the Strength of the X∙∙∙N Interaction

**DOI:** 10.3390/molecules22112034

**Published:** 2017-11-22

**Authors:** Gabriel J. Buralli, Andre N. Petelski, Nélida M. Peruchena, Gladis L. Sosa, Darío J. R. Duarte

**Affiliations:** 1 Laboratorio de Estructura Molecular y Propiedades (LEMYP), Departamento de Química, Facultad de Ciencias Exactas y Naturales y Agrimensura, Universidad Nacional del Nordeste, Avenida Libertad 5460, Corrientes 3400, Argentina; gajebu@hotmail.com (G.J.B.); arabeshai@yahoo.com.ar (N.M.P.); glaurasosa@yahoo.com.ar (G.L.S.); 2 Grupo de Investigación en Química Teórica y Experimental (QUITEX), Departamento de Ingeniería Química, Facultad Regional Resistencia, Universidad Tecnológica Nacional, French 414, Resistencia 3500, Argentina; andrepetelski@gmail.com; 3 Instituto de Química Básica y Aplicada del Nordeste Argentino (IQUIBA-NEA), UNNE-CONICET, Avenida Libertad 5460, Corrientes 3400, Argentina

**Keywords:** halogen bond, covalence, multiple bonds, QTAIM, IQA scheme

## Abstract

In the present work an in depth deep electronic study of multicenter XBs (FX)_n_/NH_3_ (X = Cl, Br and n = 1–5) is conducted. The ways in which X∙∙∙X lateral contacts affect the electrostatic or covalent nature of the X∙∙∙N interactions are explored at the CCSD(T)/aug-cc-pVTZ level and in the framework of the quantum theory of atoms in molecules (QTAIM). Calculations show that relatively strong XBs have been found with interaction energies lying between −41 and −90 kJ mol^−1^ for chlorine complexes, and between −56 and −113 kJ mol^−1^ for bromine complexes. QTAIM parameters reveal that in these complexes: (i) local (kinetics and potential) energy densities measure the ability that the system has to concentrate electron charge density at the intermolecular X∙∙∙N region; (ii) the delocalization indices [δ(A,B)] and the exchange contribution [*V*_EX_(X,N)] of the interacting quantum atoms (IQA) scheme, could constitute a quantitative measure of the covalence of these molecular interactions; (iii) both classical electrostatic and quantum exchange show high values, indicating that strong ionic and covalent contributions are not mutually exclusive.

## 1. Introduction

Halogen bonds (XBs) has been attracting more attention over the past decade. They have been generally described as R–X∙∙∙B species, in which B is an electron donor (a Lewis base) and X is the halogen atom (F, Cl, Br or I). It is known that X can form a great variety of interactions that are well-documented in the literature [1,2].

Due to the anisotropy of the electron density of halogen, when it is covalently attached, it can act both as donor and acceptor of charge density at the same time, i.e., as a Lewis base and Lewis acid, respectively [3,4]. Halogens can also interact with π electrons of aromatic systems forming the well-known aromatic XBs [5,6,7], which may play a fundamental role in the design of new drugs [8]. Actually, other species including radicals [9], metal hydrides [10,11], carbenes [12,13] are also taken as the electron donors in XBs. Furthermore, intramolecular XBs were also found to play a key role in structural chemistry [14,15].

Recently, Oliveira et al. presented a quantitative description of the intrinsic strength of XB. These authors considered from weak electrostatic interactions, with stabilization energies lower than 40 kJ mol^−1^ up to fully covalent 3c-4e interactions such as [F∙∙∙Cl∙∙∙F]^−^, with stabilization energies up to 180 kJ mol^−1^. Based on relative bond strength order (*n*) and the local X∙∙∙B stretching force constant (*k^a^*) they classed as weak predominantly electrostatic XBs (0.05 < *n*(XB) < 0.2), normal XBs (0.2 < *n*(XB) < 0.3), and strong, predominantly covalent XBs (0.3 < n(XB) < 0.6) [16].

The nature of XB has been widely discussed and is still an active subject of theoretical [2,4,17,18,19,20] and experimental chemistry research [21,22]. Most of these works aim to quantify the driving forces of association in the systems studied through different decomposition schemes of the interaction energy. However, most of these energy decomposition analysis (EDA) methods are not free of controversy, fundamentally because there exist many possible ways in which to decompose the interaction energy [23].

The application of the quantum theory of atoms in molecules (QTAIM) [24,25,26] proposed by Bader is based on the topological analysis of the distribution of electron charge density, being this a real physical property of the system. Recently, we have made several studies on the nature of some XBs [4,27,28,29,30] within the framework of this theory. These researches have shown that the electrostatic forces and charge transfer play a substantial role in the stabilization and determination of the optimal geometry of these complexes. In addition, through the topology of *L*(**r**) = −∇^2^*ρ*(**r**) function was shown that hole-lump interactions are the driving forces of these interactions [31]. Moreover, in the framework of this theory Blanco et al. proposed an energy decomposition scheme that splits the total energy exhaustively into atomic contributions, both intra- and interatomic denominated interacting quantum atoms (IQA) [32]. They demonstrated that many qualitative ideas about the chemical bond can be quantified using this scheme when it is applied to diatomic molecules [32,33]. Recently IQA analysis has been used to investigate the nature of different types of beryllium bonds [34] and XBs [35]. For the XBs FBr···NH_3_, CH_2_CHBr···NH_3_ and FBr···OH^−^ in general, electrostatically attractive force in nature were shown. However, the quantum exchange between the main atoms can never be neglected and it may constitute the dominant contribution at the equilibrium geometry [35].

Despite the extensive research carried out, the nature of the XBs continues being the center of a heated debate between theoretical chemists [19,20,28,35,36,37,38,39]. In the present work, multicenter halogen bonds (FX)_n_/NH_3_ (with X = Cl, Br and *n* = 1–5) are studied with the aim of giving answers to the following questions:

1. Can an analysis based on local kinetic and potential energies derivatives of QTAIM model contribute to the understanding of these XBs?
2. Are delocalization indices and exchange contribution of the IQA scheme a measure of the covalence of these molecular interactions, like in conventional chemical bonds?

Finally, this work seeks to the contribute to the understanding of XBs based on mecano-quantum indicators, with well-defined physical meanings.

## 2. Computational Details

Calculations were performed using the Gaussian 03 suite of programs [40]. The geometries of all complexes and the corresponding isolated compounds were fully optimized using the Møller-Plesset second-order perturbation theory [41] (MP2) with the aug-cc-pVTZ basis set. The minimum energy nature of the optimized structures was verified using the vibrational frequency analysis. Interaction energies (Equation (1)) were obtained by single point calculations using a more refined method CCSD(T) [42] with the aug-cc-pVTZ basis set. The basis set superposition error (BSSE) was also corrected by the counterpoise procedure of Boys and Bernardi [43]:

(1) $$E_{\mathrm{Int}}\left(X\cdot \cdot \cdot N\right)=E_{\mathrm{Complex}}-E_{NH_{3}}-E_{{\left(XF\right)}_{n}}+\mathrm{BSSE}$$

Topological analysis of electron density was performed with the AIMAll program using the wave functions generated from the MP2/aug-cc-pVTZ calculations. The IQA method [32] have been performed at HF/aug-cc-pVTZ level. This wavefunction allows us to analyze the pure exchange contribution (without dispersive term).

## 3. Results and Discussion

### 3.1. Geometric, Energetic, and Electron Charge Density Analysis

**Figure 1:**
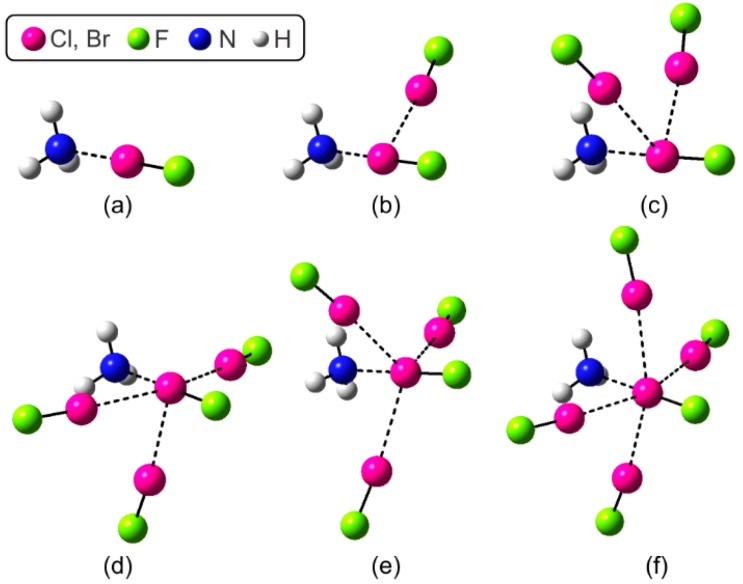
Molecular structures of complexes studied: (**a**) FX/NH_3_ (C_3v_); (**b**) (FX)_2_/NH_3_ (C_s_); (**c**) (FX)_3_/NH_3_ (C_s_); (**d**) (FX)_4_/NH_3_ (C_s_); (**e**) (FX)_4_/NH_3_ (C_3v_) and (**f**) (FX)_5_/NH_3_ (C_s_).

Figure 1 shows the molecular structures of the complexes studied. The formation of (FX)_n_/NH_3_ complexes result from the combined occurrence of lateral X∙∙∙X contacts and the central X∙∙∙N interactión. In these multicenter halogen-bonded complexes a central halogen plays a dual role of Lewis acid and base at the same time.

Table 1 reports the *E*_Int_(X∙∙∙N) and some relevant geometrical parameters. It can be seen the presence of XBs of considerable strength, compared with FCl∙∙∙PH_2_OH (*E*_Int_ = −54.8 kj mol^−1^) and FCl∙∙∙PF_2_H (*E*_Int_ = −29.9 kj mol^−1^) complexes calculated at CCSD(T)/aug-cc-pVTZ level, recently considered by Oliveira et al. as strong and predominantly covalent XBs [16].

The interaction energies lie between −41 and −90 kJ mol^−1^ for chlorine complexes, and between −56 and −113 kJ mol^−1^ for bromine complexes. As expected, *E*_Int_(X∙∙∙N) increase in magnitude in the order FX/NH_3_ < (FX)_2_/NH_3_ < (FX)_3_/NH_3_ < (FX)_4_/NH_3_ < (FX)_5_/NH_3_ (for the same X) and they are consistent with the intermolecular distances X∙∙∙N. It can be clearly seen that the X∙∙∙N bond strengthens with the increasing number of XF molecules more than 10 kJ mol^−1^ for each molecule are added. The energy of (FX)_5_/NH_3_ being double of that FX/NH_3_. A very good linear relationships between number of XF molecules and *E*_Int_(X∙∙∙N) was found (R^2^ = 0.9990 and 0.9912 for (FCl)_n_/NH_3_ and (FBr)_n_/NH_3,_ respectively).

These multicenter halogen bonds present the typical geometrical characteristics of XBs. The bond angle of F‒X_central_···N interaction is close to 180° and the intermolecular distances are shorter than the sum of the van der Waals radii of the X and N atoms, Δ*d*_vdW_(X∙∙∙N) > 0. These can be interpreted as the distance of penetration of X and N atoms electronic densities, and they are consistent with the calculated interaction energies.

The X···N intermolecular distances are comparable to those reported on other XBs Cl···N [28,29] and Br···N [29,35]. Oliveira et al. have reported an intermolecular distance of 2.320 Å for the same complex at CCSD(T)/aug-cc-pVTZ level [16]. Legon et al. have also experimentally studied the FCl/NH_3_ system the gas phase by rotational spectroscopy. In their work, they found a *d*_Cl···N_ = 2.376 Å and suggested that the [H_3_NCl]^+^∙∙∙F^−^ ion pair should be taken into account in order to explain both the high force constant obtained and the valence bond description of the complex [45]. The determined symmetry shown by these researchers is in agreement with the results presented here. However, the N···Cl length is longer than that obtained from theoretical calculations. It is important to note that the unchanged monomer geometries have been considered to evaluate the spectroscopic geometry of this complex.

The X–F bond of the central molecule is elongated in all complexes, Δ*d*(X–F) > 0. This elongation in (FCl)_n_/NH_3_ complexes increases gradually as the X∙∙∙N interaction becomes stronger, while in (FBr)_n_/NH_3_ complexes relatively little variation is observed. The stretching of these bonds can be understood through the analysis of the electron transfer. The formation of a XB (R–X∙∙∙B) is accompanied by electron transfer from B to the σ*_(X–R)_ antibonding orbital. According to Hobza et al. [46] the elongation of the R–X bond is caused by this electron transfer. While, when the R–X bond is shortened the electron transfer to σ*_(X–R)_ is only of minor importance [46]. It is clear that a deep analysis of orbital interactions is necessary to understand why the X–F bond elongates for chlorine complexes and not for the bromine ones. However, in the present work we focused on the X∙∙∙N bond itself and its covalence; therefore a more detailed analysis of this phenomenon is beyond the scope of this paper.

The analysis of the local topological properties at the X∙∙∙N and X∙∙∙X bond critical points (BCP), such as the electron charge density, *ρ*(**r**_b_), and its Laplacian function, ∇^2^*ρ*(**r**_b_) present values typical of closed-shell interactions (*ρ*(**r**_b_) are relatively low and ∇^2^*ρ*(**r**_b_) > 0) (see Table S1).

The fact that the X∙∙∙N interaction is progressively strengthened with the addition of XF units is a situation that allows us to inquire about possible descriptors of the strength of the X∙∙∙N interaction. Although it is true that FX∙∙∙NH_3_ (X = Cl, Br) complexes had already been studied experimentally [45,47] and theoretically [2,28,29,35,48,49], to the best of our knowledge, this grade of coordination in the framework of XBs has never been explored. There is only one work where the central halogen is involved as a halogen donor and it is combined simultaneously with a maximum of three hydrogen bonds [50]. In this work the authors have demonstrated that the combined halogen and hydrogen bonding within complexes of FBr, NCH, and FH can give rise to a series of strongly bound complexes.

### 3.2. Local Kinetic and Potential Energies Analysis

The role played by the electron kinetic energy [51,52] and the electron potential energy [53,54] in the formation of chemical bonding has been under discussion for many years. Latterly, Bacskay et al. [55] have stressed the fundamental role of the kinetic energy in the description of covalent bonding and Bader [56] emphasized that chemical bonding is “a result of the lowering of the potential energy in the bonding region caused by the accumulation of density that attracts the nuclei”.

The local kinetic energy density, *G*(**r**_b_), and the local potential energy density, *V*(**r**_b_), might be used to analyze the electronic behavior at the intermolecular BCP. In addition, *V*(**r**_b_) can be decomposed into two terms, according to (Equation (2)):

*V*(**r**_b_) = *V*_en_(**r**_b_) + *V*_rep_(**r**_b_) (2)

The attractive part of *V*(**r**_b_) is *V*_en_(**r**_b_) = −*ρ*(**r**_b_). *V*_nuc_(**r**_b_) (in which *V*_nuc_(**r**_b_) is the potential from the nuclei at (**r**_b_) and the implicit repulsive part consists of both inter-electronic repulsion, *V*_ee_(**r**_b_), and nuclear-nuclear repulsion, *V*_nn_(**r**_b_), contributions. Table 2 reports local kinetic density and potential energy density and their components derivatives of QTAIM.

It is observed that, *G*(**r**_b_) and |*V*(**r**_b_)| are increased with an increase of the stabilization energy, *E*_stab_(X∙∙∙N) (in which *E*_stab_(X∙∙∙N) = −*E*_int_(X∙∙∙N)). Figure S1 shows for each set of interactions Cl···N and Br···N, linear relationships between *G*(**r**_b_) and *E*_stab_(X∙∙∙N) and between |*V*(**r**_b_)| and *E*_stab_(X∙∙∙N). In addition, very good linear relationships between *G*(**r**_b_) and *ρ*(**r**_b_) and between |*V*(**r**_b_)| and *ρ*(**r**_b_) are observed (see Figure S2). It appears that *G*(**r**_b_) and *V*(**r**_b_) might be considered as a measure of the strength of the interaction and of the ability of the system to concentrate electron charge density at the intermolecular X∙∙∙N BCP. This interpretation is in partial agreement with Espinosa et al. [57]. These authors, in the framework of hydrogen bonds, state that *V*(**r**_b_) represents the capacity of the system to concentrate electrons at the intermolecular BCP, while *G*(**r**_b_) shows the tendency of the system to dilute electrons at the same point.

**Figure 2:**
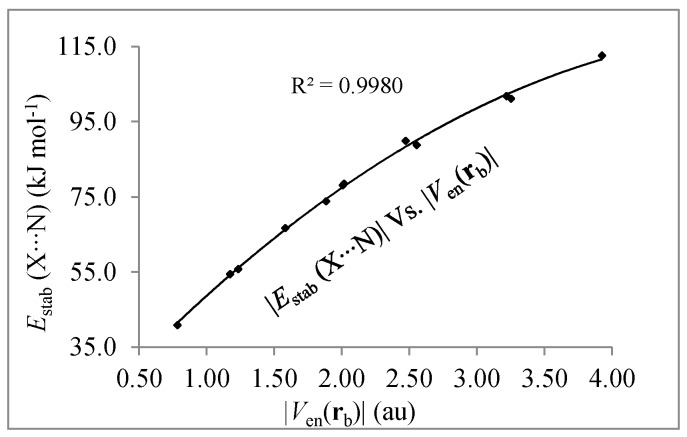
Correlation between *E*_stab_(X∙∙∙N) and |*V*_en_(**r**_b_)|.

Further, we have found important relationships between the interaction energies and the components of the potential energy density. These values are reported in Table 2 and it is observed that for each set of interactions Cl···N and Br···N, in general terms, all of them follow the same trend as interaction energies. However, the relationship between *E*_stab_(X∙∙∙N) and |*V*_en_(**r**_b_)| deserves special attention. The *V*_en_(**r**_b_) is a measure of the electrostatic force exerted by the nuclear charges of X and N on the electron charge density at the X∙∙∙N BCP region. In Figure 2 a good quadratic relationship between these parameters is observed, and they do not depend on the pair of interacting atoms (Cl∙∙∙N/Br∙∙∙N). Similar relationships have been found in F_n_X∙∙∙CO (n = 1, 3, 5 and X = Cl, Br, I) [30] and R–X∙∙∙X–R (with R = −H, −Cl, −F and X = Cl, Br, I) [58] complexes. Therefore, the electrostatic interaction between the electron charge density localized at the intermolecular region and the nuclear charge of the interacting atoms plays a significant role in stabilizing these complexes.

In the context of QTAIM there are basically three local magnitudes that allow discussing about the nature of the molecular interactions, electron charge density, *ρ*(**r**_b_), the *L*(**r**_b_) = −∇^2^*ρ*(**r**_b_) function (Equation (3)) and the local electronic energy density, *H*(**r**_b_) (Equation (4)):

−*L*(**r**_b_) = ¼∇^2^*ρ*(**r**_b_) = 2*G*(**r**_b_) + *V*(**r**_b_) (3)

*H*(**r**_b_) = *G*(**r**_b_) + *V*(**r**_b_) (4)

While these magnitudes have a well-defined physical meaning, the joint interpretation of these quantities in the context of molecular interactions is not free of controversy. There are several researchers who consider that a negative value of *H*(**r**_b_) is associated with the covalence of the interaction [59,60,61,62]. However, we have a different interpretation of the meaning of *H*(**r**_b_) negativity. In previous works we studied XBs and HBs complexes in order to understand the nature of such interactions [27,63,64]. These works have concluded that “the decrease in *H*(**r**_b_) with the interaction strengthening observed in the HBs as well as the XBs, is mainly due to the increase in the attractive electrostatic part of the interaction energy and in lesser extent to the increase in its covalent character, as it is commonly considered”. A more detailed analysis of the components of *L*(**r**_b_) and *H*(**r**_b_), bearing in mind the Equation (2), reveals that *L*(**r**_b_) > 0 and *H*(**r**_b_) < 0 provided that |*V*_en_(**r**_b_)| > 2*G*(**r**_b_) + *V*_rep_(**r**_b_) and |*V*_en_(**r**_b_)| > *G*(**r**_b_) + *V*_rep_(**r**_b_), respectively. That is, the only term that becomes positive and negative to *L*(**r**_b_) and *H*(**r**_b_), respectively is *V*_en_(**r**_b_), whose physical meaning is well-defined. It appears that, *V*_en_(**r**_b_) is an appropriate local parameter to measure the attractive electrostatic part of the X∙∙∙N interaction.

### 3.3. Localization and Delocalization Indices

The localization [λ(A)] and delocalization [δ(A,B)] indices provide a clear indication of the progression of bonding from ionic to polar to covalent [56]. The λ(A) accounts for the number of electrons localized within atomic basin A and δ(A,B) is a measure of the number of electrons that are shared or exchanged between A and B independently of the nature of the interaction [65]. These magnitudes are reported in Table 3 and could be used to inquire about the covalence of the X∙∙∙N interactions. In this sense, it has been shown that the degree of sharing of electrons decreases with the increasing polar nature of the bonds (cf. in species CO to CN^−^ to NO^+^) [65].

In Table 3 it is observed that to each set of interactions Cl∙∙∙N and Br∙∙∙N, the number of shared electrons [δ(X,N)] increases, while the number of localized electrons [λ(X) and λ(N)] decreases with the strengthening of the X∙∙∙N interaction (see Table 1).

**Figure 3:**
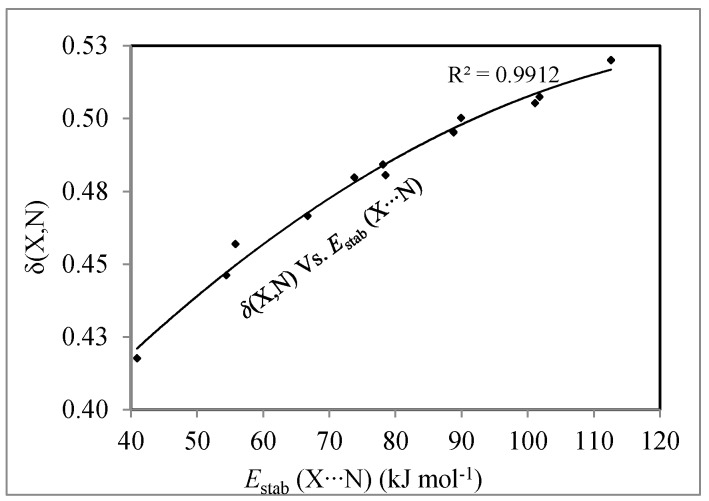
Relationships between *E*_stab_(X∙∙∙N) and δ(X,N).

Figure 3 shows quadratic relationships between the *E*_stab_(X∙∙∙N) and δ(X,N), independently of the pair of interacting atoms. Therefore, the number of electrons that are shared or exchanged between X and N plays an important role in the stability of complexes and it appears that is a good measure of covalence of these interactions.

### 3.4. IQA Analysis

The IQA scheme [32] constitutes a rigorous tool to inquire about the physical nature of the inter-atomic (X∙∙∙N) interaction. According to this partitioning scheme the inter-atomic interaction energy can be decomposed into:

*E*_Int_(X,N) = *V*_nn_(X,N) + *V*_en_(X,N) + *V*_en_(N,X) + *V*_ee,C_(X,N) + *V*_ee,EX_(X,N) (5)

The first four terms correspond to classical Coulombic electrostatic interactions [*V*_C_(X,N)] and the last term is exchange contribution [*V*_EX_(X,N)], provided that the wavefunction is calculated at Hartree-Fock level. Therefore, the inter-atomic interaction energy can be written as:

*E*_Int_(X,N) = *V*_C_(X,N) + *V*_EX_(X,N) (6)

These values are collected in Table 4. The inter-atomic interaction energy between main atoms is highly stabilizing and ranges between −0.2231 au (FCl/NH_3_) and −0.3247 au [(FBr)_5_/NH_3_)]. Very good linear relationships have been found between *E*_Int_(X,N) calculated by IQA and *E*_Int_(X∙∙∙N) calculated using super-molecule approximation (R^2^ = 0.9877 and 0.9969 for (FCl)_n_/NH_3_ and (FBr)_n_/NH_3_, respectively).

Many qualitative ideas about the chemical bond can be quantified using this scheme of decomposition [32]. The component *V*_C_(X,N) provides valuable information about electrostatic interactions (ionic bonds), while *V*_EX_(X,N) term gives account of covalence between two atoms derived from the Pauli exclusion principle [32]. When we compare these magnitudes, it is observed that they are predominantly electrostatic in nature *V*_C_(X,N) > *V*_EX_(X,N). However, the quantum contributions (covalence) [*V*_EX_(X,N)] cannot be ignored in fact they also make a significant contribution (between 34.3% and 44.7%) to total interaction energy. Therefore, the antisymmetric nature of the wave function that allows the exchange of electrons between atoms is also important for the stabilizing of these complexes.

Moreover, the stabilizing electrostatic components *V*_en_(X,N) and *V*_en_(N,X) are increased in magnitude in the sense FX/NH_3_ < (FX)_2_/NH_3_ < (FX)_3_/NH_3_ < (FX)_4_/NH_3_ < (FX)_5_/NH_3_ and in all the complexes |*V*_en_(X,N)| < |*V*_en_(N,X)| is observed. Therefore, the interaction between the electron cloud of the N atom (Lewis base) and the nuclear charge of the halogen atom (Lewis acid) is the driving force in these complexes. That is, IQA analysis supports the idea that XBs are basically Lewis acid-base interactions [66].

On the other hand, as mentioned earlier the delocalization index represents the number of electrons exchanged or shared between X and N atoms and the inter-atomic exchange energy is an energetic measure of this exchange. Indeed there is a relationship between both magnitudes [67]:

(7) $$V_{\mathrm{EX}}(X,N)\approx -\frac{\delta (X,N)}{2d(X\cdot \cdot \cdot N)}$$

This equation to some extent relates to the parameters involved in the definition of covalent bond given by IUPAC, which establishes that a covalent bond occurs when “a region of relatively high electron density between nuclei which arises at least partly from sharing of electrons and gives rise to an attractive force and characteristic internuclear distance” [68]. Thus, a large value for δ(A,B) and small *d*(X∙∙∙N) correspond to a large absolute value for the inter-atomic exchange energy and a considerable covalent character of the X∙∙∙N interaction. The values calculated with this expression are in a very good agreement with those calculated by IQA scheme (R^2^ = 0.9977).

It is important to note that both *V*_Cl_(X,N) and *V*_EX_(X,N) components increase in magnitude with the strengthening of the X∙∙∙N interactions and the concomitant decrease of the X∙∙∙N intermolecular distance. However, as more XF units are added to the central X, the exchange term increases more rapidly in magnitude than the classical electrostatic contribution. This is reflected in the percentages with which they contribute to the inter-atomic interaction energy, %EX and %Cl respectively. It appears that the classical interactions favor the approach between interacting atoms and these, in turn, favour the electronic exchange. This last observation has also been made by Syzgantseva et al. [35], according to these authors in the XBs FBr···NH_3_, CH_2_CHBr···NH_3_ and FBr···OH^−^ at long-range, electrostatic interactions clearly dominate (as expected from the σ-hole model) and is responsible for the initiation of the bond formation process. While at the equilibrium geometry, exchange is an important contribution to the complex stabilization once it is formed [35]. According to the previous discussion both classical electrostatic and quantum exchange show high values, indicating that strong ionic and covalent contributions in these halogen bonds are not mutually exclusive.

**Figure 4:**
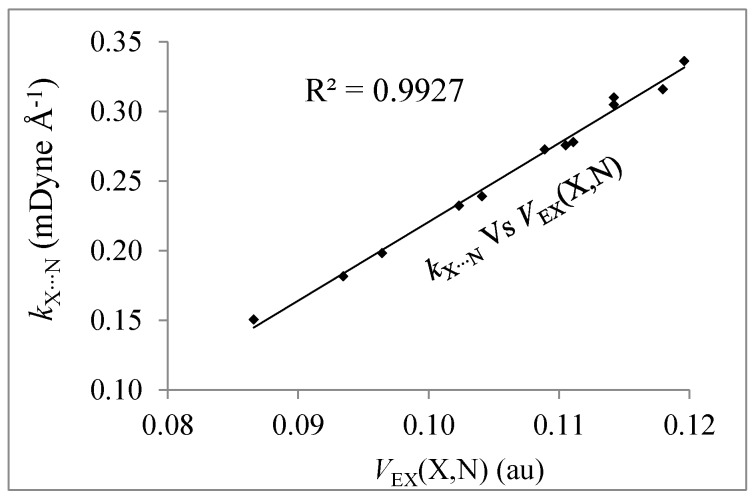
Relationships between *V*_EX_(X,N) and *k*_X∙∙∙N_.

On the other hand, the force constants can be interpreted as a measure of the strength of the chemical bonds and *V*_EX_(X,N) as a measure of covalence. Figure 4 shows a very good linear correlation between force constants of the X∙∙∙N bond (*k*_X∙∙∙N_) and *V*_EX_(X,N) component. Therefore, the covalence measured through electronic exchange is a very good descriptor of the strength of the X∙∙∙N interactions.

Finally, the covalence is not an observable magnitude in the sense of quantum mechanics. However, its use is deeply rooted in the language of chemists, so it is necessary to have a bridge between this concept and some quantum-mechanical observable magnitudes. It appears that, magnitudes that have a well-defined physical meaning, such as *δ*(A,B) and *V*_EX_(A,B) can shed light on the covalence of the molecular interactions as it occurs with in diatomic molecules.

### 3.5. Molecular Electrostatic Potentials

**Figure 5:**
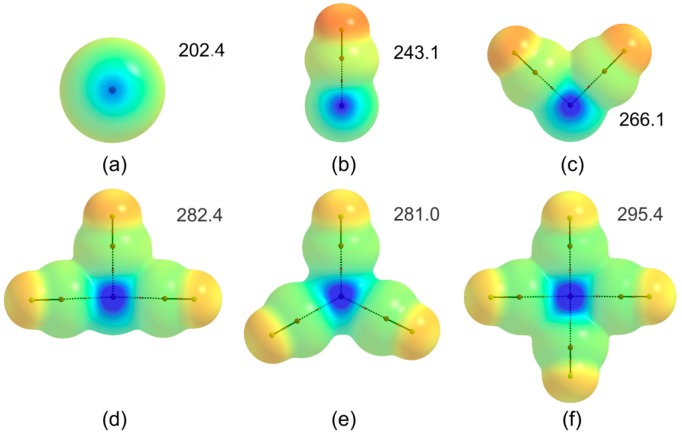
Molecular electrostatic potential on the 0.001 au electron density isosurface of the (BrF)_n_ supermolecules. The maximum (*V*_S,max_) electrostatic potential (in kJ mol^−1^) on the central Br atom is indicated. The equivalent values for (ClF)_n_ set of complexes are: (**a**) 167.3; (**b**) 200.7; (**c**) 221.1; (**d**) 237.1; (**e**) 237.1; (**f**) 251.8. The values of the MEP vary between −105 kJ mol^−1^ (red) and +295 kJ mol^−1^ (blue).

The molecular electrostatic potential (MEP) is a real physical property very useful for the molecular recognition [69]. In order to analyze the electrostatic behavior on the central halogen X, the σ-hole magnitude (*V*_S,max_) has been computed over supermolecules without considering ammonia. Results are shown in Figure 5. In all superstructures, *V_S,_*_max_ is found on central halogen which interacts with LP of NH_3_.

As expected*,* as more XF units are added to the central X, *V_S,_*_max_ values increase in magnitude (see Figure 5). This fact reflects the electron-withdrawing capacity of the lateral XF molecules. It is interesting to note that a good linear relationship is established between *E*_stab_(X···N) and *V_S,_*_max_ (see Figure S3), independently of the pair of interacting atoms (Cl∙∙∙N/Br∙∙∙N). Therefore, the electrostatic interactions play a substantial role in the stabilization of these complexes.

The fact that *V*_S,max_ values increase in magnitude in the sense FX < (FX)_2_ < (FX)_3_ < (FX)_4_ < (FX)_5_ leads us to think that in the same way, the ionic character of X∙∙∙N interaction increases. That is, the σ-hole model predicts that ionic character of the X∙∙∙N interaction increase in the same sense that covalence (according to IQA model) does. These observations have attracted attention, because it is accepted that the forces involved in the formation of the XBs are primarily electrostatic [39,70].

## 4. Conclusions

In this work, an energy analysis was carried out at CCSD(T)/aug-cc-pVTZ level, together with: (i) electron charge density analysis; (ii) local (kinetics and potential) energy densities analysis; (iii) localization and delocalization indexes; (iv) IQA decomposition analysis, among others, in (FX)_n_/NH_3_ (with X = Cl, Br and *n* = 1–5) complexes. It was performed to see the ways in which X∙∙∙X lateral contacts affect the electrostatic or covalent nature of the X∙∙∙N interactions.

Calculations show that the X∙∙∙N XB is strongly reinforced by multiple X∙∙∙X lateral contacts (interaction energies lying between −41 and −90 kJ mol^−1^ for chlorine complexes, and between −56 and −113 kJ mol^−1^ for bromine complexes).

The local energy densities, *V*(**r**_b_) and *G*(**r**_b_) measure the ability that the system has to concentrate electron charge density at the intermolecular X∙∙∙N BCP. The electrostatic part of the X∙∙∙N interaction can be scanned in terms of the local attractive term of the potential energy density, *V*_en_(**r**_b_).

The delocalization indices [δ(A,B)] and exchange contribution [*V*_EX_(X,N)] of the IQA scheme, could constitute a quantitative measure of the covalence of these molecular interactions. In addition, the covalence measured through electronic exchange is a very good descriptor of the strength of the X∙∙∙N interactions measured through force constants (*k*_X∙∙∙N_).

According to IQA model, the classical contribution (ionic bond or electrostatic interaction) plays a key role in stabilizing these complexes. However, the quantum contribution (covalence) cannot be ignored, it also makes a significant aid (between 34.3% and 44.7%) to total interaction energy.

## Figures and Tables

**Table 1:** Selected geometric parameters and interaction energies.

Complexes ^a^	*d*(X∙∙∙N)	Δ*d*_vdW_(X∙∙∙N)	Δ*d*(X‒F)	*E*_Int_(X∙∙∙N)
FCl/NH_3_ (C_3v_)	2.272	0.441	0.074	−40.9
(FCl)_2_/NH_3_ (C_s_)	2.224	0.489	0.080	−54.4
(FCl)_3_/NH_3_ (C_s_)	2.191	0.521	0.086	−66.7
(FCl)_4_/NH_3_ (C_s_)	2.165	0.548	0.093	−78.1
(FCl)_4_/NH_3_ (C_3v_)	2.166	0.547	0.092	−78.5
(FCl)_5_/NH_3_ (C_s_)	2.141	0.571	0.100	−89.9
FBr/NH_3_ (C_3v_)	2.325	0.542	0.067	−55.8
(FBr)_2_/NH_3_ (C_s_)	2.281	0.587	0.066	−73.8
(FBr)_3_/NH_3_ (C_s_)	2.252	0.616	0.066	−88.8
(FBr)_4_/NH_3_ (C_s_)	2.230	0.638	0.067	−101.8
(FBr)_4_/NH_3_ (C_3v_)	2.231	0.636	0.068	−101.1
(FBr)_5_/NH_3_ (C_s_)	2.212	0.655	0.072	−112.6

^a^ Symmetry point group are indicated. *d*(X∙∙∙N): intermolecular distance. Δ*d*_vdW_(X∙∙∙N): is the difference between the equilibrium intermolecular distances and the sum of the van der Waals radii [44] of X and N atoms. Δ*d*(X–F): is the change of X–F bond length of the XF central upon complex formation. *E*_Int_(X∙∙∙N): interaction energy calculated at CCSD(T)/aug-cc-pVTZ and corrected by BSSE. Distances in angstrom, angles in degrees and energies in kJ mol^−1^.

**Table 2:** Local kinetic energy density and potential energy density and its components at the intermolecular X∙∙∙N BCP. ^a^

Complexes ^b^	*G*(r_b_)	*V*(r_b_)	*V*_en_(r_b_)	*V*_rep_(r_b_)
FCl/NH_3_ (C_3v_)	0.0425	−0.0471	−0.7817	0.7346
(FCl)_2_/NH_3_ (C_s_)	0.0470	−0.0550	−1.1712	1.1162
(FCl)_3_/NH_3_ (C_s_)	0.0502	−0.0613	−1.5793	1.5180
(FCl)_4_/NH_3_ (C_s_)	0.0529	−0.0669	−2.0067	1.9398
(FCl)_4_/NH_3_ (C_3v_)	0.0528	−0.0669	−2.0148	1.9479
(FCl)_5_/NH_3_ (C_s_)	0.0553	−0.0724	−2.4701	2.3977
FBr/NH_3_ (C_3v_)	0.0415	−0.0490	−1.2320	1.1830
(FBr)_2_/NH_3_ (C_s_)	0.0456	−0.0567	−1.8833	1.8266
(FBr)_3_/NH_3_ (C_s_)	0.0484	−0.0626	−2.5532	2.4906
(FBr)_4_/NH_3_ (C_s_)	0.0504	−0.0671	−3.2172	3.1501
(FBr)_4_/NH_3_ (C_3v_)	0.0506	−0.0676	−3.2513	3.1838
(FBr)_5_/NH_3_ (C_s_)	0.0522	−0.0715	−3.9234	3.8519

^a^ Local topological parameters were determined at the X∙∙∙N intermolecular BCP. ^b^ Symmetry point group are indicated. *G*(**r**_b_): local kinetic energy density. *V*(**r**_b_): local potential energy density. *V*_en_(**r**_b_): electron-nuclear attractive contribution to virial field. *V*_rep_(**r**_b_): repulsive contribution to virial field (inter-electronic repulsion and nuclear-nuclear repulsion). All values in atomic units.

**Table 3:** Electronic localization and delocalization indices.

Complexes	λ(Ω)		δ(X,N)
	X	N	
FCl/NH_3_ (C_3v_)	16.070	6.865	0.419
(FCl)_2_/NH_3_ (C_s_)	15.963	6.839	0.446
(FCl)_3_/NH_3_ (C_s_)	15.885	6.821	0.467
(FCl)_4_/NH_3_ (C_s_)	15.833	6.809	0.484
(FCl)_4_/NH_3_ (C_3v_)	15.818	6.796	0.481
(FCl)_5_/NH_3_ (C_s_)	15.782	6.793	0.500
FBr/NH_3_ (C_3v_)	33.958	6.880	0.457
(FBr)_2_/NH_3_ (C_s_)	33.746	6.863	0.480
(FBr)_3_/NH_3_ (C_s_)	33.609	6.851	0.495
(FBr)_4_/NH_3_ (C_s_)	33.531	6.844	0.508
(FBr)_4_/NH_3_ (C_3v_)	33.505	6.832	0.505
(FBr)_5_/NH_3_ (C_s_)	33.475	6.830	0.520

λ(Ω): localization indices of X and N basins. δ(X,N): delocalization indices between X and N basins.

**Table 4:** IQA interatomic contribution in the X∙∙∙N interactions.

Complexes	*E*_int_(X,N)	*V*_nn_(X,N)	*V*_en_(X,N)	*V*_en_(N,X)	*V_ee,C_*(X,N)	*V_EX_*(X,N)	*V*_C_(X,N)	%EX	%Cl
FCl/NH_3_ (C_3v_)	−0.2231	27.7198	−26.8930	−32.4325	31.4691	−0.0866	−0.1365	38.8	61.2
(FCl)_2_/NH_3_ (C_s_)	−0.2380	28.3166	−27.4573	−33.1334	32.1326	−0.0964	−0.1416	40.5	59.5
(FCl)_3_/NH_3_ (C_s_)	−0.2482	28.7381	−27.8627	−33.6362	32.6167	−0.1041	−0.1441	41.9	58.1
(FCl)_4_/NH_3_ (C_s_)	−0.2565	29.0902	−28.2074	−34.0642	33.0360	−0.1111	−0.1454	43.3	56.7
(FCl)_4_/NH_3_ (C_3v_)	−0.2554	29.0769	−28.1955	−34.0363	33.0101	−0.1105	−0.1449	43.3	56.7
(FCl)_5_/NH_3_ (C_s_)	−0.2640	29.4118	−28.5250	−34.4562	33.4234	−0.1179	−0.1461	44.7	55.3
FBr/NH_3_ (C_3v_)	−0.2722	55.7605	−54.7218	−65.5421	64.3247	−0.0934	−0.1787	34.3	65.7
(FBr)_2_/NH_3_ (C_s_)	−0.2955	56.8462	−55.7332	−66.9047	65.5985	−0.1023	−0.1932	34.6	65.4
(FBr)_3_/NH_3_ (C_s_)	−0.3094	57.5746	−56.4298	−67.8420	66.4967	−0.1089	−0.2005	35.2	64.8
(FBr)_4_/NH_3_ (C_s_)	−0.3181	58.1413	−56.9833	−68.5692	67.2073	−0.1142	−0.2039	35.9	64.1
(FBr)_4_/NH_3_ (C_3v_)	−0.3174	58.1007	−56.9483	−68.5426	67.1870	−0.1142	−0.2032	36.0	64.0
(FBr)_5_/NH_3_ (C_s_)	−0.3247	58.5986	−57.4442	−69.2236	67.8642	−0.1196	−0.205	36.8	63.1

*E*_Int_(X,N): inter-atomic interaction energy. *V*_nn_(X,N): repulsion energy between nuclear charge of atom X and nuclear charge of atom N. *V*_en_(X,N): attraction energy between nuclear charge of atom N and electron density distribution of atom X. *V*_en_(N,X): attraction energy between nuclear charge of atom X and electron density distribution of atom N. *V_ee,C_*(X,N): Coulomb part of two-electron interaction energy between atom X and atom N. *V_EX_*(X,N): exchange part of two-electron interaction energy between atom X and atom N. *V*_C_(X,N) = *V*_nn_(X,N) + *V*_en_(X,N) + *V*_en_(N,X) + *V*_ee,C_(X,N). All energetic values in atomic units.

## Supplementary Materials

Supplementary materials are available online. Table S1: Local topological properties of the electron charge density at the X∙∙∙N and X∙∙∙X interactions BCP, Figure S1: Correlation of *E*_stab_(X∙∙∙N)| with |*V*(**r**_b_)| and *G*(**r**_b_), Figure S2: Correlation of *ρ*(**r**_b_) (X∙∙∙N) with |*V*(**r**_b_)| and *G*(**r**_b_), and Figure S3: Correlation between *V_S_*_,max_ and *E*_stab_(X∙∙∙N).
