# Supplementary material for: Multicenter (FX)n/NH3 Halogen Bonds (X = Cl, Br and n = 1–5). QTAIM Descriptors of the Strength of the X∙∙∙N Interaction
Source: Molecules. 2017 Nov 22;22(11):2034. doi: 10.3390/molecules22112034 (PMC6150306; doi:10.3390/molecules22112034)
Supplement: Supplementary file 1 [file molecules-22-02034-s001.pdf]

Supporting Information

Multicenter halogen bond $(FX)_n/NH_3$ (X= Cl, Br and n=1-5). QTAIM descriptors of the strength of the X···N interaction

Gabriel J. Buralli[1,3], Andre N. Petelski,[2,3] Nélida M. Peruchena,[1,3] Gladis L. Sosa[1,2,3] Darío J. R. Duarte[1,3,*]

[1] Laboratorio de Estructura Molecular y Propiedades (LEMYP), Departamento de Química, Facultad de Ciencias Exactas y Naturales y Agrimensura, Universidad Nacional del Nordeste, Avenida Libertad 5460 (3400), Corrientes, Argentina; gajebu@hotmail.com, arabeshai@yahoo.com.ar, glaurasosa@yahoo.com.ar, djr_duarte@hotmail.com
[2] Grupo de Investigación en Química Teórica y Experimental (QUITEX), Departamento de Ingeniería Química, Facultad Regional Resistencia, Universidad Tecnológica Nacional, French 414 (H3500CHJ), Resistencia, Chaco, Argentina. andrepetelski@gmail.com, glaurasosa@yahoo.com.ar
[3] Instituto de Química Básica y Aplicada del Nordeste Argentino (IQUIBA-NEA), UNNE-CONICET, Avenida Libertad 5460, 3400 Corrientes, Argentina; gajebu@hotmail.com, andrepetelski@gmail.com, arabeshai@yahoo.com.ar, glaurasosa@yahoo.com.ar, djr_duarte@hotmail.com

* Corresponding authors:
Darío J. R. Duarte, e-mail: djr_duarte@hotmail.com, Phone/Fax: +54 379 4473930

Contents

Pg. S2 **Table S1.** Local topological properties of the electron charge density at the X···N and X···X interactions BCP.

Pg. S2 **Figure S1.** Correlation of E_{stab}(X···N)| with $|V(\mathbf{r}_b)|$ and $G(\mathbf{r}_b)$.

Pg. S2 **Figure S2.** Correlation of $\rho(\mathbf{r}_b)$ (X···N) with $|V(\mathbf{r}_b)|$ and $G(\mathbf{r}_b)$.

Pg. S3 **Figure S3.** Correlation between $V_{S,max}$ and E_{stab}(X···N).

Table S1. Local topological properties of the electron charge density at the X···N and X···X interactions BCP.

Complexes[a)]	X···N			X···X[b)]		
	$\rho(\mathbf{r}_b)$	$\nabla^2\rho(\mathbf{r}_b)$	$H(\mathbf{r}_b)$	$\rho(\mathbf{r}_b)$	$\nabla^2\rho(\mathbf{r}_b)$	$H(\mathbf{r}_b)$
FCl/NH_3 (C_{3v})	0.0567	0.1520	-0.0045	-	-	-
$(FCl)_2/NH_3$ (C_s)	0.0642	0.1563	-0.0079	0.0191	0.0691	0.0024
$(FCl)_3/NH_3$ (C_s)	0.0701	0.1566	-0.0111	0.0180	0.0657	0.0023
(FCl)4/NH3 (C_s)	0.0753	0.1553	-0.0140	0.0164	0.0606	0.0022
$(FCl)_4/NH_3$ (C_{3v})	0.0752	0.1550	-0.0141	0.0170	0.0627	0.0023
$(FCl)_5/NH_3$ (Cs)	0.0803	0.1523	-0.0172	0.0158	0.0588	0.0022
FBr/NH_3 (C_{3v})	0.0569	0.1359	-0.0075	-	-	-
$(FBr)_2/NH_3$ (C_s)	0.0640	0.1375	-0.0112	0.0258	0.0702	0.0007
$(FBr)_3/NH_3$ (C_s)	0.0693	0.1364	-0.0143	0.0234	0.0667	0.0010
$(FBr)_4$/NH3 (C_s)	0.0733	0.1345	-0.0167	0.0206	0.0612	0.0140
$(FBr)_4$/NH3 (C_{3v})	0.0736	0.1343	-0.0170	0.0216	0.0633	0.0146
$(FBr)_5$/NH3 (C_s)	0.0771	0.1319	-0.0193	0.0188	0.0572	0.0129

a) Symmetry point group are indicated. b) Average values. $\rho(\mathbf{r}_b)$: electron density. $\nabla^2\rho(\mathbf{r}_b)$: Laplacian of the electron density. $H(\mathbf{r}_b)$: total electronic energy density. All values in atomic units.

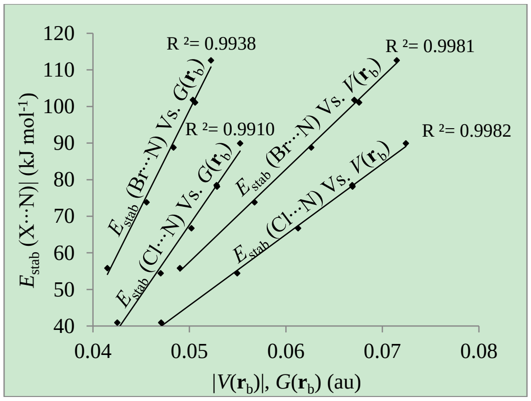

Figure S1. Correlation of E_{stab}(X···N)| with $|V(\mathbf{r}_b)|$ and $G(\mathbf{r}_b)$.

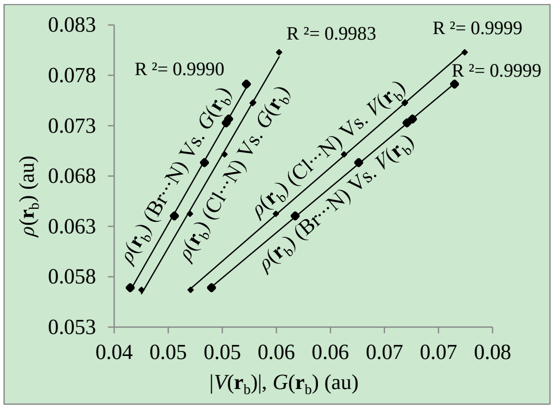

Figure S2. Correlation of $\rho(\mathbf{r}_b)$ (X···N) with $|V(\mathbf{r}_b)|$ and $G(\mathbf{r}_b)$.

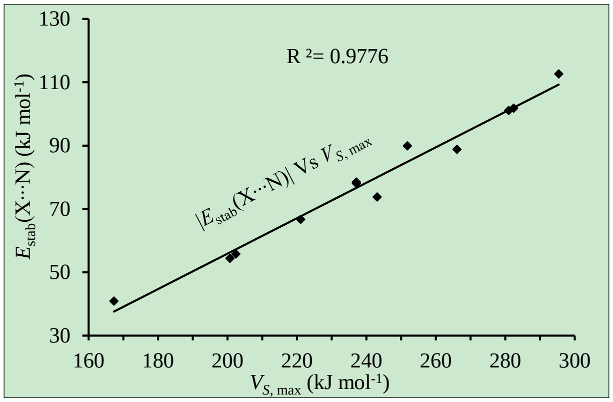

Figure S3. Linear relationship between $V_{S,\text{max}}$ and E_{stab}(X···N).
